# Prognostic Impact of Neutropenia in Cancer Patients with Septic Shock: A 2009–2017 Nationwide Cohort Study

**DOI:** 10.3390/cancers14153601

**Published:** 2022-07-24

**Authors:** Sang-Min Kim, Youn-Jung Kim, Ye-Jee Kim, Won-Young Kim

**Affiliations:** 1 Department of Emergency Medicine, Ulsan University College of Medicine, Asan Medical Center, Seoul 05505, Korea; swdarkhorse@gmail.com (S.-M.K.); yjkim.em@gmail.com (Y.-J.K.); 2 Department of Clinical Epidemiology and Biostatistics, Asan Medical Center, Seoul 05505, Korea; kimyejee@amc.seoul.kr

**Keywords:** septic shock, neutropenia, cancer patients, prognostic impact

## Abstract

**Simple Summary:**

The prognostic impact of neutropenia on mortality in cancer patients with septic shock remains controversial despite recent advances in cancer and sepsis management. This study aimed to determine whether neutropenia could be related to an increase in short-term and long-term mortality. This population-based, case–control study used data from the National Health Insurance Service of Korea. Adult cancer patients who presented to the emergency department with septic shock from 2009 to 2017 were analyzed. The 30-day and 1-year mortality rates were evaluated as short-term and long-term outcomes. After adjustment for confounders, neutropenia was independently associated with decreased 30-day and 1-year mortality rates. Neutropenia did not increase mortality in cancer patients with septic shock, suggesting that neutropenia may not be used as a single triage criterion for withholding intensive care in cancer patients presenting to the emergency department with septic shock.

**Abstract:**

(1) Background: Neutropenia’s prognostic impact on mortality in cancer patients with septic shock remains controversial despite recent advances in cancer and sepsis management. This population-based, case–control study aimed to determine whether neutropenia could be related to an increase in short-term and long-term mortality. (2) Methods: This population-based, case–control study used data from the National Health Insurance Service of Korea. Adult cancer patients who presented to the emergency department with septic shock from 2009 to 2017 were included. The 30-day and 1-year mortality rates were evaluated as short-term and long-term outcomes. Cox proportional hazard regression was performed after adjusting for age, sex, Charlson comorbidity index, and neutropenia. (3) Results: In 43,466 adult cancer patients with septic shock, the 30-day and 1-year mortality rates were 52.1% and 81.3%, respectively. In total, 6391 patients had neutropenic septic shock, and the prevalent cancer type was lung cancer, followed by leukemia, non-Hodgkin’s lymphoma, stomach cancer, and colon cancer. Furthermore, 30-day and 1-year mortality was lower in patients with neutropenia than in those without neutropenia. After adjustment for confounders, neutropenia was independently associated with decreased 30-day and 1-year mortality rates. (4) Conclusions: In cancer patients presenting to the emergency department with septic shock, the presence of neutropenia did not increase mortality. This suggests that neutropenia may not be used as a single triage criterion for withholding intensive care in cancer patients presenting to the emergency department with septic shock.

## 1. Introduction

Septic shock is a common cause of intensive care unit (ICU) admission and is associated with higher mortality in patients with malignancies [1,2]. Neutropenia, defined as an absolute neutrophil count (ANC) of <500/mm^3^, is a frequent side effect of chemotherapeutic agents. However, the prognostic impact of neutropenia on sepsis and septic shock has not been thoroughly evaluated. Intensivists had commonly associated neutropenia with higher mortality in critically ill patients, leading to reluctance in life-saving therapies for these patients [3]. However, the overall survival rate has improved due to advances in the management of sepsis and cancer, such as prompt administration of empiric, broad-spectrum antibiotics and antifungals based on the recent guidelines [4,5]. Moreover, previous studies including a significant number of patients with neutropenia have suggested that timely admission of cancer patients to the ICU is essential for survival [4,6,7] and that the adjusted influence of neutropenia on the mortality of cancer patients with critical illness is not statistically significant [8]. Therefore, neutropenia may not be a driving criterion for admitting cancer patients to the ICU, withdrawing life-sustaining therapies in these populations.

However, limited data are available, particularly regarding patients with neutropenia developing septic shock, one of the most critical complications in cancer patients. Therefore, a current guideline for neutropenic septic patients with cancer called for additional studies without excluding these specific populations [9]. To better understand the prognostic impact of neutropenia on the outcome in septic shock patients with cancer, we conducted a population-based study using data from the National Health Insurance Service (NHIS) of Korea. This study aimed to determine whether neutropenia would be related to an increase in long-term and short-term mortality in cancer patients with septic shock.

## 2. Materials and Methods

### 2.1. Study Design and Data Source

This population-based cohort study used data from the Korean National Health Information Database (NHID) that was collected between 2009 and 2017 and released in 2019. The NHIS requires all Korean citizens to register for national healthcare insurance through the enactment of the Medical Insurance Act in 1963 [10]. The Korean NHIS is responsible for maintaining and managing the NHID, a public database covering health care utilization, health screening, socio-demographic variables, and mortality of all Korean citizens. The data cover almost all Koreans (approximately 50 million individuals) and the clinical data from all healthcare facilities in Korea [11]. We extracted data on demographic information, medical bill details, medical treatments, disease histories, and prescriptions, which were converted as insurance claim information for the first day of medical treatment. However, laboratory and radiologic data were not available from the NHID.

The primary outcome of this study was all-cause 30-day mortality, and the secondary outcome was all-cause 1-year mortality. All cancer patients with septic shock in our study population were followed up from the index date to 1 year or until death, if it occurred before 1 year. This study was approved by the Institutional Review Board of the Asan Medical Center (Study number: 2019-0743) and by the NHIS inquiry commission. The personal privacy of the study participants was protected through de-identification of the national insurance claims data.

### 2.2. Study Patients and Data Definitions

We selected all patients admitted to a hospital through the emergency department (ED) who fulfilled the clinical surveillance definition of septic shock. The Third International Consensus Definition for Sepsis and Septic Shock (Sepsis-3) defined septic shock as “life-threatening organ dysfunction caused by a dysregulated host response to infection, requiring vasopressor therapy, and a known elevated lactate level.” [12]. We used a clinical surveillance definition of septic shock based on concurrent vasopressors, antibiotics, and blood cultures [13]. Among patients with a blood culture order and concomitant administration of intravenous antibiotics (suspected infection), those who received vasopressors, including dopamine, norepinephrine, epinephrine, vasopressin, and phenylephrine, were considered to have septic shock.

We identified patients with cancer among the initial screened cohort as those having hospital visits with a cancer diagnosis code within the preceding 90 days of their septic shock hospitalization, according to the International Classification of Diseases, 10th edition (ICD-10) (C00-C97) and rare, incurable disease registration code (V193, V027) simultaneously to minimize misclassification. The accuracy of identifying cancer patients using the combination of diagnosis codes in the NHID was similar to that in the Korea National Cancer Incidence Database, estimated to be 98.2% complete [14,15]. Furthermore, to extract patients with neutropenia, we used the ICD-10 diagnosis code for neutropenia (D70) or prescription information for granulocyte colony-stimulating factor (G-CSF) at admission. The underlying comorbidities were identified using ICD-10 codes when two or more hospital visits with the relevant diagnostic codes within a year prior to the septic shock date were recorded, and the Charlson comorbidity index (CCI) was calculated. We excluded patients aged <18 years during their septic shock hospitalization or those without complete data. In cases where patients visited more than once because of septic shock, we used the data collected at the first admission.

### 2.3. Statistical Analysis

Categorical variables are presented as numbers with percentages, and continuous variables are presented as means and standard deviations. Hazard ratios (HRs) and 95% confidence intervals (CIs) for all-cause 30-day and 1-year mortality were estimated using Cox proportional hazard regression analyses. After adjustment for age, sex, and CCI, the adjusted HRs of the hospitalization year on 30-day and 1-year mortality were calculated. All the tests of significance used two-sided *p* values < 0.05. These analyses were conducted using Enterprise Guide version 7.1 (SAS Institute Inc., Cary, NC, USA).

## 3. Results

### 3.1. Study Population

**Figure 1:**
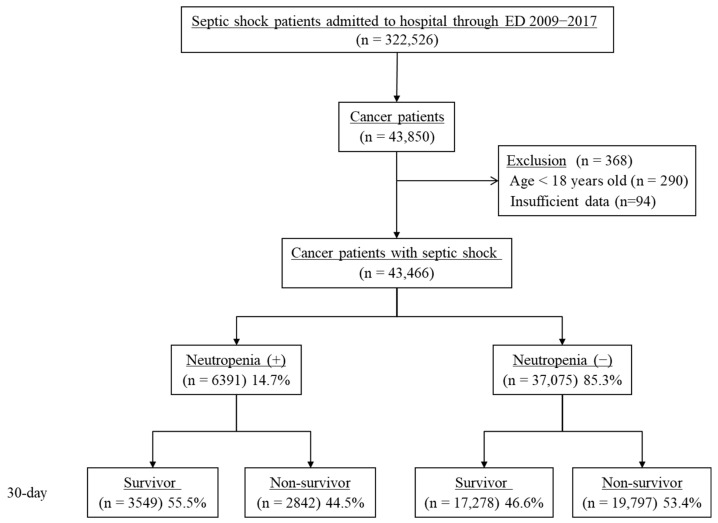
Patient flow diagram. ED, emergency department.

Among the 322,526 septic shock patients admitted to the hospital through the ED from 2009 to 2017, 43,850 patients were identified as having cancer with diagnosis codes according to ICD-10 (C00-C97, V193, V027) within the preceding 90 days of their septic shock hospitalization (Figure 1). After excluding patients aged <18 years (n = 290) and those without sufficient data (n = 94), we finally analyzed 43,466 patients. Neutropenia was identified in 6391 (14.7%) patients with the diagnosis code for neutropenia and prescription information of G-CSF. The 30-day mortality rates of neutropenic and non-neutropenic septic shock patients were 44.5% and 53.4%, respectively.

### 3.2. Baseline and Clinical Characteristics of the Study Population

Table 1 presents the characteristics of the study patients according to the status of neutropenia. The mean age was older in non-neutropenic patients than in neutropenic patients (67.8 ± 12.6 vs. 62.6 ± 12.9; *p* < 0.001). Among comorbidities, hypertension, diabetes, congestive heart failure, and liver cirrhosis were significantly frequent in patients without neutropenia than in those with neutropenia. The mean CCI was not significantly different between both groups. However, the composition of the cancer subtype was significantly different between both groups. Hematologic malignancies, such as leukemia, non-Hodgkin’s lymphoma, and multiple myeloma were more frequent in patients with neutropenia than in those without neutropenia. In contrast, liver, colon, gall bladder, and pancreatic cancers were more frequent in patients without neutropenia than in those with neutropenia.

Both chemotherapy and radiotherapy were applied more to patients with neutropenia than to those without neutropenia within the 30-day and 90-day time-point. The overall 30-day and 1-year mortality was 52.1% and 81.3%, respectively. Patients with neutropenia showed better survival than those without neutropenia at the 30-day and 1-year time-points.

### 3.3. Mortality Rate of Septic Shock According to Cancer Subtype and Prevalence Rate of Neutropenic Septic Shock According to Chemotherapy Status

**Figure 2:**
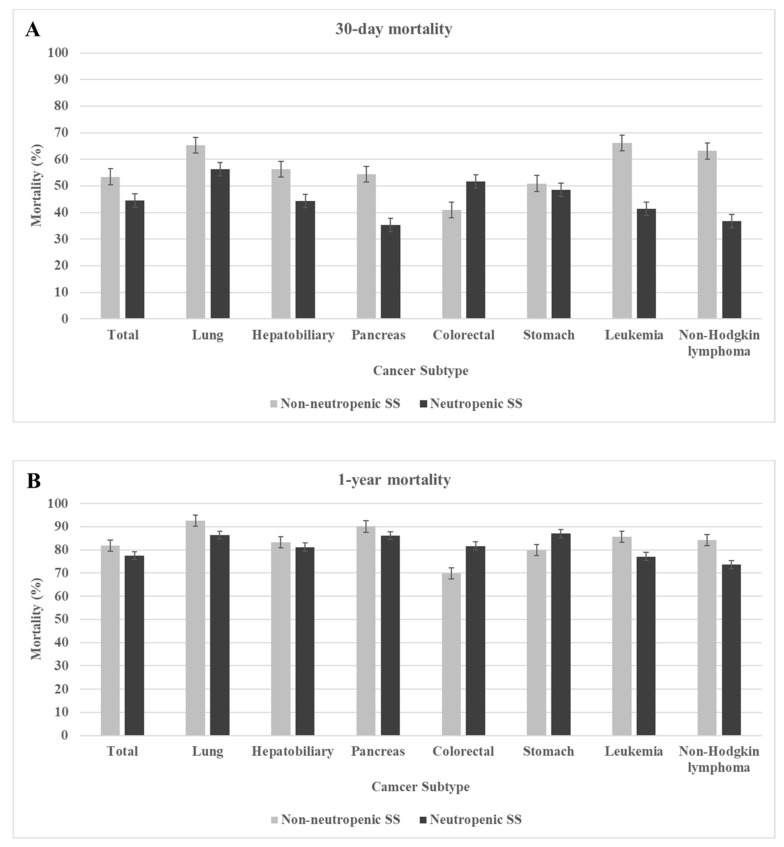
Mortality rate of septic shock according to cancer subtype. (**A**) 30-day mortality rate according to cancer subtype; (**B**) 1-year mortality rate according to cancer subtype. SS, septic shock.

Figure 2 presents the mortality rate of septic shock in each cancer subtype. The 30-day mortality rate was significantly higher in non-neutropenic septic shock than in neutropenic septic shock in all cancer subtypes, except the colorectal subtype. The 1-year mortality rate was significantly higher in non-neutropenic septic shock than in neutropenic septic shock in lung cancer, leukemia, and non-Hodgkin lymphoma. In hepatobiliary and pancreatic cancer, non-neutropenic septic shock patients showed a higher 1-year mortality rate than neutropenic septic shock patients, although without statistical significance. In contrast, in colorectal and stomach cancer, neutropenic septic shock patients showed a higher 1-year mortality rate than non-neutropenic septic shock patients.

**Figure 3:**
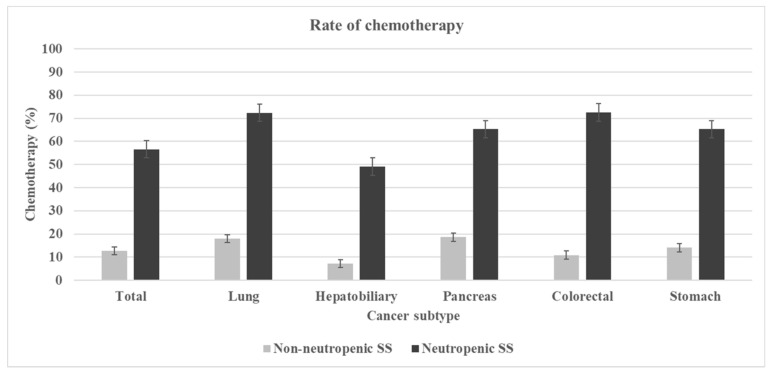
Comparison of rate of chemotherapy within preceding 30 days in each group according to cancer subtype. SS, septic shock.

The prevalence rate of neutropenic septic shock according to the chemotherapy status within 30 days is presented in Figure 3. In all cancer subtypes, neutropenic septic shock was more frequent in patients treated with chemotherapy than in those not treated with chemotherapy.

### 3.4. Factors Associated with 30-Day and 1-Year Mortality in Septic Shock Survivors

Multivariate-adjusted analysis was performed to identify the potential risk factors associated with 30-day and 1-year mortality, including variables such as age, sex, CCI, and neutropenia (Table 2). Neutropenia was independently associated with a decreased 30-day (HR 0.811, 95% CI 0.779–0.844; *p* < 0.001) and 1-year (HR 0.861, 95% CI 0.836–0.888; *p* < 0.001) mortality rate after adjustment for other confounders. Table S1 showed an adjusted hazard ratio of neutropenia in 30-day and 1-year mortality in septic shock patients according to the definition of neutropenia and study population (with or without hematologic malignancy). It showed a consistently significant relationship between neutropenia and mortality of septic shock patients.

## 4. Discussion

The main finding of this study was that approximately 15% of the cancer patients with septic shock had neutropenia, and it was associated with decreased 30-day and 1-year mortality after adjusting with confounders, such as age, sex, and CCI.

We previously reported the mortality trends of septic shock in cancer patients with claims data using diagnostic codes [16]. We used a clinical surveillance definition of septic shock based on concurrent vasopressors, antibiotics, and blood cultures. However, Rhee et al. reported that only positive blood culture findings along with increased serum lactate levels could improve the sensitivity of severe sepsis/septic shock detection using clinical claims data [13]. In this study, we extracted data on patients who had claims data of “blood culture order” from the national insurance claims data without extracting data on blood culture results. As not all the septic shock patients show positive blood culture results [17], it is reasonable to include all patients with culture order than those with positive blood culture results to reflect reality. Moreover, Kadri et al. demonstrated that clinical surveillance definitions for septic shock were superior for identifying septic shock over claims data using the “septic shock” code through a clinical medical record review [18]. Although there could be a limitation in identifying septic shock patients, the definition in this study might be reasonable.

In this study, we used the operational definition of neutropenic septic shock using the diagnosis code for neutropenia or administration information of G-CSF after admission in these populations. Previous studies used the diagnosis code for neutropenia or agranulocytosis for identifying patients with neutropenia [19,20,21,22]. Additionally, Weycker et al. reported that patients with neutropenic fever could be identified using claims data with a positive predictive value > 80% with the diagnosis code [23]. However, in actual practice, many patients often do not receive neutropenia as the principal diagnostic code; therefore, there could be a possibility that only a limited number of cases would be identified. In Korea, health insurance covers the administration of G-CSF for therapeutic use only when patients treated with chemotherapy have agranulocytosis (ANC < 500 cells/mm^3^) or neutropenia (ANC < 1000 cells/mm^3^) with fever. Therefore, a previous study used the prescription information of G-CSF for identifying patients with febrile neutropenia in the Korean NHIS [24]. Therefore, we further included patients who received G-CSF for therapeutic use for identifying neutropenic septic shock patients; moreover, it might be practical due to the scrutiny of the NHIS.

Neutropenia remains a common side effect of most treatments administered to cancer patients [25]. The reported proportion of neutropenic sepsis in cancer patients ranges from 7% to 45%, depending on study characteristics, such as patient selection [1,26,27]. In this study, 14.7% of septic shock patients were identified to have neutropenia. Although patients with neutropenia are susceptible to infection due to a deficiency in innate immune systems and an association with other complications [28,29,30], neutropenia as a prognostic factor in cancer patients with sepsis remains controversial. Reilly et al. reported that neutropenic sepsis was independently associated with a higher risk of acute kidney injury, but not with 30-day mortality [31]. Furthermore, a previous study investigating 464 septic shock patients admitted to the oncologic ICU showed no difference in mortality between the neutropenic and non-neutropenic groups [32]. This study showed that neutropenia was independently associated with decreased mortality after adjusting for potential confounders. To the best of our knowledge, this study is the first to investigate the prognostic impact of neutropenia in cancer patients with septic shock using population-based claims data. As the discrepancies in previous reports about the prognosis and incidence of neutropenic sepsis might be due to admission policies and patient selection, the strength of this study is that it included the claims data of all cancer patients and did not exclude patients by specific criteria. It might be beneficial to add valuable evidence to assess neutropenia as prognostic factors in cancer patients with septic shock.

This study showed improved survival in patients with neutropenia. However, this finding does not imply that neutropenic patients treated with G-CSF have better outcomes. Instead, it implies that neutropenia, which is commonly considered a poor prognostic factor, does not influence the mortality of cancer patients with septic shock. The reason for the decreased mortality in neutropenic septic shock patients might depend on their baseline performance status. Patients with neutropenia were younger and had fewer comorbidities than patients without neutropenia. Furthermore, patients in the early stages of cancer might more often receive chemotherapy, resulting in neutropenia, than those in the later stages. A previous study with critically ill patients with cancer showed that patients with neutropenia related to chemotherapy have better survival than patients without neutropenia [33].Considering the treatment status, such as chemotherapy and radiotherapy, there could be a possibility that the disease status of patients with neutropenia was not significantly more advanced than that of patients without neutropenia. Previous studies showed that the primary determinant of the outcome in critically ill cancer patients was the baseline performance status with accompanying organ failure [34,35]. Furthermore, Vincent et al. suggested that the risk factors for 120-day mortality after ICU admission in patients with solid tumors were the type of cancer, systemic extension of the disease, and need for invasive mechanical ventilation, vasopressors, or renal replacement therapy [36].

Notably, among patients with colon and stomach cancer, those with neutropenic septic shock showed higher 1-year mortality than those without neutropenic septic shock. An abdominal syndrome, such as neutropenic enterocolitis, is an uncommon but life-threatening complication that could affect the prognosis [37,38]. Therefore, the susceptible mechanism of infection in such a type of cancer might be fatal in patients with neutropenia. Moreover, there could be a possibility that different chemotherapy regimens depending on the cancer subtype might affect the prognosis of neutropenic septic shock.

Although we included the patients treated with G-CSF to identify neutropenia, the role of G-CSF in neutropenic sepsis is controversial. G-CSF can increase peripheral blood leukocyte and lymphocyte cell counts [39]. The current guidelines recommend prophylactic use of G-CSF for patients treated with chemotherapy with the risk of developing febrile neutropenia [40,41]. However, administration of G-CSF at the time of neutropenic sepsis is not supported by sufficient evidence [42]. The primary role of administering G-CSF in patients with cancer is to prevent neutropenia and maintain the dose intensity of chemotherapy.

Due to the recent advances in cancer treatment and sepsis management, the overall survival rate of cancer patients with septic shock has improved [43]. Moreover, appropriate selection of the patients to be admitted to the ICU would be helpful for a better outcome [34,44]. The recent guidelines for ICU admission in patients with cancer suggest that the classical predictors of mortality are not relevant, and the triage criteria usually used are unreliable [45]. In neutropenia, mixed results were reported due to a potential selection bias by physicians in providing treatments. This study suggests that it is not appropriate to include neutropenia in the triage criteria for intensive care and predict mortality in patients with cancer with septic shock. Further studies are needed to confirm this finding.

This study has some limitations. First, given the inherent methodological limitation of a nationwide observation study, the potential impact due to confounding factors would be significant, making it hard to generalize. Second, the NHID in Korea did not provide specific laboratory data such as neutrophil counts and serum lactate levels, which are essential for the definition of septic shock and neutropenia. We identified patients with operational definitions using diagnosis codes which proved its activity in identifying neutropenic patients in previous studies. Furthermore, we used prescription information of G-CSF to identify neutropenic patients, which is a broader definition of neutropenia than previous studies. Although the sensitivity would increase with this effort, the potential impact of misclassification was unavoidable. In addition, if we used another definition of neutropenia, the results would be different. Third, cancer details, such as stage, treatment settings, and performance status, were missing. The possible explanation for the better survival in neutropenic patients could be speculation of the disease status from the treatment status; this information would be necessary and could be a significant confounder, although it is not included in the Cox model. However, we included age, sex, and CCI, confounders for which the most reliable and robust information was available. The results could change if other factors were included in the model. Fourth, the NHID in Korea did not provide specific laboratory and clinical data, which might affect the outcomes. Data about sepsis management, such as fluid administration, antibiotics, and vasopressors, would be helpful.

## 5. Conclusions

We observed that neutropenia did not increase mortality in septic shock patients, suggesting that neutropenia may not be used as a single triage criterion for withholding intensive care in cancer patients with septic shock presenting to the ED.

## Figures and Tables

**Table 1:** Baseline and clinical characteristics of septic shock patients with and without neutropenia.

Characteristics	All Patients (n = 43,466)	Non-Neutropenic SS (n = 37,075)	Neutropenic SS (n = 6391)	*p*-Value
Age, years	67.0 ± 12.7	67.8 ± 12.6	62.6 ± 12.9	<0.001
Sex, male	28,067 (64.6)	24,166 (65.2)	3901 (61.0)	<0.001
Comorbidities				
Hypertension	23,136 (53.2)	20,091 (54.2)	3045 (47.7)	<0.001
Diabetes mellitus	16,977 (39.1)	14,758 (39.8)	2219 (34.7)	<0.001
Congestive heart failure	5745 (13.2)	5008 (13.5)	737 (11.5)	<0.001
Chronic lung disease	5008 (11.5)	4301 (11.6)	707 (11.1)	0.213
Renal failure	2783 (6.4)	2397 (6.5)	386 (6.0)	0.199
Liver cirrhosis	4974 (11.4)	4705 (12.7)	269 (4.2)	<0.001
CCI (mean)	6.30 ± 3.89	6.29 ± 3.91	6.36 ± 3.80	0.186
CCI (subgroup)				
0–2	7816 (18.0)	6709 (18.1)	1107 (17.3)	
3–4	9652 (22.2)	8160 (22.0)	1492 (23.4)	
5–7	8256 (19.0)	7231 (19.5)	1025 (16.0)	
8+	17,742 (40.8)	14,975 (40.4)	2767 (43.3)	
Cancer type				<0.001
Brain	772 (1.8)	736 (2.0)	36 (0.6)	
Lung	6657 (15.3)	5646 (15.2)	1011 (15.8)	
Liver	6238 (14.4)	6074 (16.4)	164 (2.6)	
Colon	4494 (10.3)	4122 (11.1)	372 (5.8)	
Stomach	3684 (8.5)	3284 (8.9)	400 (6.3)	
Gall bladder	1981 (4.6)	1911 (5.2)	70 (1.1)	
Pancreas	1943 (4.5)	1799 (4.9)	144 (2.3)	
Leukemia	1917 (4.4)	1,053 (2.8)	864 (13.5)	
Non-Hodgkin’s lymphoma	1475 (3.4)	724 (2.0)	751 (11.8)	
Female reproductive system	1249 (2.9)	862 (2.3)	387 (6.0)	
Breast	1112 (2.6)	752 (2.0)	360 (5.6)	
Kidney/bladder	1095 (2.5)	1010 (2.7)	85 (1.3)	
Multiple myeloma	923 (2.1)	635 (1.7)	288 (4.5)	
Male reproductive system	754 (1.7)	641 (1.7)	113 (1.8)	
Oropharynx	439 (1.0)	364 (1.0)	75 (1.2)	
Esophagus	391 (0.9)	324 (0.9)	67 (1.0)	
Thyroid	169 (0.4)	160 (0.4)	9 (0.1)	
Larynx	149 (0.3)	131 (0.4)	18 (0.3)	
Hodgkin lymphoma	50 (0.1)	28 (0.1)	22 (0.3)	
Other, unspecified	2995 (6.9)	2564 (6.9)	431 (6.7)	
Multiple	4979 (11.5)	4255 (11.5)	724 (11.3)	
Treatment				
Radiotherapy within 30 days	1607 (3.7)	1272 (3.4)	335 (5.2)	<0.001
Chemotherapy within 30 days	8310 (19.1)	4693 (12.7)	3617 (56.6)	<0.001
Radiotherapy within 90 days	3723 (8.6)	3019 (8.1)	704 (11.0)	<0.001
Chemotherapy within 90 days	13,831 (31.8)	9425 (25.4)	4406 (68.9)	<0.001
Outcome				
30-day mortality	22,639 (52.1)	19,797 (53.4)	2842 (44.5)	<0.001
1-year mortality	35,325 (81.3)	30,369 (81.9)	4956 (77.5)	<0.001

Values are expressed as the mean ± standard deviation or number (%). CCI, Charlson comorbidity index; SS, septic shock.

**Table 2:** Factors associated with 30-day and 1-year mortality in septic shock survivors according to multivariate Cox proportional hazard regression analysis.

Characteristics	30-Day Mortality			1-Year Mortality		
	Adjusted HR	95% CI	*p*-Value	Adjusted HR	95% CI	*p*-Value
Neutropenia	0.811	0.779–0.844	<0.001	0.861	0.836–0.888	<0.001
Age	1.007	1.006–1.008	<0.001	1.009	1.008–1.010	<0.001
Female sex	0.919	0.894–0.945	<0.001	0.919	0.899–0.939	<0.001
CCI						
0–2	Reference			Reference		
3–4	1.356	1.296–1.419	<0.001	1.312	1.267–1.359	<0.001
5–7	1.431	1.366–1.499	<0.001	1.405	1.355–1.456	<0.001
8+	1.840	1.767–1.915	<0.001	1.862	1.805–1.921	<0.001

HR, hazard ratio; CI, confidential interval; CCI, Charlson comorbidity index.

## Data Availability

No new data were created or analyzed in this study. Data sharing is not applicable to this article.

## Supplementary Materials

The following are available online at https://www.mdpi.com/article/10.3390/cancers14153601/s1, Table S1: An adjusted hazard ratio of neutropenia in 30-day and 1-year mortality in septic shock survivors according to the definition.

## References

[1] Azoulay E., Mokart D., Pène F., Lambert J., Kouatchet A., Mayaux J., Vincent F., Nyunga M., Bruneel F., Laisne L.M. (2013). Outcomes of critically ill patients with hematologic malignancies: Prospective multicenter data from France and Belgium—a groupe de recherche respiratoire en réanimation onco-hématologique study. J. Clin. Oncol..

[2] Soares M., Bozza F.A., Azevedo L.C., Silva U.V., Corrêa T.D., Colombari F., Torelly A.P., Varaschin P., Viana W.N., Knibel M.F. (2016). Effects of Organizational Characteristics on Outcomes and Resource Use in Patients with Cancer Admitted to Intensive Care Units. J. Clin. Oncol..

[3] Georges Q., Azoulay E., Mokart D., Soares M., Jeon K., Oeyen S., Rhee C.K., Gruber P., Ostermann M., Hill Q.A. (2018). Influence of neutropenia on mortality of critically ill cancer patients: Results of a meta-analysis on individual data. Crit. Care.

[4] Azoulay E., Pène F., Darmon M., Lengliné E., Benoit D., Soares M., Vincent F., Bruneel F., Perez P., Lemiale V. (2015). Managing critically Ill hematology patients: Time to think differently. Blood Rev..

[5] Mokart D., Pastores S.M., Darmon M. (2014). Has survival increased in cancer patients admitted to the ICU? Yes. Intensive Care Med..

[6] de Montmollin E., Tandjaoui-Lambiotte Y., Legrand M., Lambert J., Mokart D., Kouatchet A., Lemiale V., Pène F., Bruneel F., Vincent F. (2013). Outcomes in critically ill cancer patients with septic shock of pulmonary origin. Shock.

[7] Mokart D., Lambert J., Schnell D., Fouché L., Rabbat A., Kouatchet A., Lemiale V., Vincent F., Lengliné E., Bruneel F. (2013). Delayed intensive care unit admission is associated with increased mortality in patients with cancer with acute respiratory failure. Leuk. Lymphoma.

[8] Bouteloup M., Perinel S., Bourmaud A., Azoulay E., Mokart D., Darmon M. (2017). Outcomes in adult critically ill cancer patients with and without neutropenia: A systematic review and meta-analysis of the Groupe de Recherche en Réanimation Respiratoire du patient d’Onco-Hématologie (GRRR-OH). Oncotarget.

[9] Kochanek M., Schalk E., von Bergwelt-Baildon M., Beutel G., Buchheidt D., Hentrich M., Henze L., Kiehl M., Liebregts T., von Lilienfeld-Toal M. (2019). Management of sepsis in neutropenic cancer patients: 2018 guidelines from the Infectious Diseases Working Party (AGIHO) and Intensive Care Working Party (iCHOP) of the German Society of Hematology and Medical Oncology (DGHO). Ann. Hematol..

[10] Cheol Seong S., Kim Y.Y., Khang Y.H., Heon Park J., Kang H.J., Lee H., Do C.H., Song J.S., Hyon Bang J., Ha S. (2017). Data Resource Profile: The National Health Information Database of the National Health Insurance Service in South Korea. Int. J. Epidemiol..

[11] Kim J.A., Yoon S., Kim L.Y., Kim D.S. (2017). Towards Actualizing the Value Potential of Korea Health Insurance Review and Assessment (HIRA) Data as a Resource for Health Research: Strengths, Limitations, Applications, and Strategies for Optimal Use of HIRA Data. J. Korean Med. Sci..

[12] Singer M., Deutschman C.S., Seymour C.W., Shankar-Hari M., Annane D., Bauer M., Bellomo R., Bernard G.R., Chiche J.D., Coopersmith C.M. (2016). The Third International Consensus Definitions for Sepsis and Septic Shock (Sepsis-3). JAMA.

[13] Rhee C., Murphy M.V., Li L., Platt R., Klompas M. (2015). Comparison of trends in sepsis incidence and coding using administrative claims versus objective clinical data. Clin. Infect. Dis..

[14] Seo H.J., Oh I.H., Yoon S.J. (2012). A comparison of the cancer incidence rates between the national cancer registry and insurance claims data in Korea. Asian Pac. J. Cancer Prev..

[15] Hong S., Won Y.J., Park Y.R., Jung K.W., Kong H.J., Lee E.S. (2020). Cancer Statistics in Korea: Incidence, Mortality, Survival, and Prevalence in 2017. Cancer Res. Treat..

[16] Kim Y.-J., Kim M.-J., Kim Y.-J., Kim W.Y. (2021). Short and Long-Term Mortality Trends for Cancer Patients with Septic Shock Stratified by Cancer Type from 2009 to 2017: A Population-Based Cohort Study. Cancers.

[17] Li Y., Guo J., Yang H., Li H., Shen Y., Zhang D. (2021). Comparison of culture-negative and culture-positive sepsis or septic shock: A systematic review and meta-analysis. Crit. Care.

[18] Kadri S.S., Rhee C., Strich J.R., Morales M.K., Hohmann S., Menchaca J., Suffredini A.F., Danner R.L., Klompas M. (2017). Estimating Ten-Year Trends in Septic Shock Incidence and Mortality in United States Academic Medical Centers Using Clinical Data. Chest.

[19] Kuderer N.M., Dale D.C., Crawford J., Cosler L.E., Lyman G.H. (2006). Mortality, morbidity, and cost associated with febrile neutropenia in adult cancer patients. Cancer.

[20] Chindaprasirt J., Wanitpongpun C., Limpawattana P., Thepsuthammarat K., Sripakdee W., Sookprasert A., Wirasorn K. (2013). Mortality, length of stay, and cost associated with hospitalized adult cancer patients with febrile neutropenia. Asian Pac. J. Cancer Prev..

[21] Kozma C.M., Dickson M., Chia V., Legg J., Barron R. (2012). Trends in neutropenia-related inpatient events. J. Oncol. Pract..

[22] Lingaratnam S., Thursky K.A., Slavin M.A., Kirsa S.W., Bennett C.A., Worth L.J. (2011). The disease and economic burden of neutropenic fever in adult patients in Australian cancer treatment centres 2008: Analysis of the Victorian Admitted Episodes Dataset. J. Intern. Med..

[23] Weycker D., Sofrygin O., Seefeld K., Deeter R.G., Legg J., Edelsberg J. (2013). Technical evaluation of methods for identifying chemotherapy-induced febrile neutropenia in healthcare claims databases. BMC Health Serv. Res..

[24] Kim D., Lee S., Youk T., Hong S. (2021). Incidence and Clinical Outcomes of Febrile Neutropenia in Adult Cancer Patients with Chemotherapy Using Korean Nationwide Health Insurance Database. Yonsei Med. J..

[25] Maschmeyer G., Beinert T., Buchheidt D., Cornely O.A., Einsele H., Heinz W., Heussel C.P., Kahl C., Kiehl M., Lorenz J. (2009). Diagnosis and antimicrobial therapy of lung infiltrates in febrile neutropenic patients: Guidelines of the infectious diseases working party of the German Society of Haematology and Oncology. Eur. J. Cancer.

[26] Soares M., Salluh J.I.F., Torres V.B.L., Leal J.V.R., Spector N. (2008). Short- and long-term outcomes of critically ill patients with cancer and prolonged ICU length of stay. Chest.

[27] Lee D.S., Suh G.Y., Ryu J.A., Chung C.R., Yang J.H., Park C.M., Jeon K. (2015). Effect of Early Intervention on Long-Term Outcomes of Critically Ill Cancer Patients Admitted to ICUs. Crit. Care Med..

[28] Kalil A.C., Opal S.M. (2015). Sepsis in the severely immunocompromised patient. Curr. Infect. Dis. Rep..

[29] Regazzoni C.J., Khoury M., Irrazabal C., Myburg C., Galvalisi N.R., O’Flaherty M., Sarquis S.G., Poderoso J.J. (2003). Neutropenia and the development of the systemic inflammatory response syndrome. Intensive Care Med..

[30] Talcott J.A., Finberg R., Mayer R.J., Goldman L. (1988). The medical course of cancer patients with fever and neutropenia. Clinical identification of a low-risk subgroup at presentation. Arch. Intern. Med..

[31] Reilly J.P., Anderson B.J., Hudock K.M., Dunn T.G., Kazi A., Tommasini A., Charles D., Shashaty M.G., Mikkelsen M.E., Christie J.D. (2016). Neutropenic sepsis is associated with distinct clinical and biological characteristics: A cohort study of severe sepsis. Crit. Care.

[32] Laserna A., Fowler C., O’Connell K., Manjappachar N., Martin P., Cuenca J., Urso C., Gutierrez C., Malik I., Erfe R. (2020). 1670: NEUTROPENIC SEPTIC SHOCK OUTCOMES IN CANCER PATIENTS AND THE INFLUENCE OF COLONY-STIMULATING FACTOR. Crit. Care Med..

[33] Souza-Dantas V.C., Salluh J.I.F., Soares M. (2011). Impact of neutropenia on the outcomes of critically ill patients with cancer: A matched case-control study. Ann. Oncol..

[34] Lemiale V., Pons S., Mirouse A., Tudesq J.J., Hourmant Y., Mokart D., Pène F., Kouatchet A., Mayaux J., Nyunga M. (2020). Sepsis and Septic Shock in Patients with Malignancies: A Groupe de Recherche Respiratoire en Réanimation Onco-Hématologique Study. Crit. Care Med..

[35] de Vries V.A., Müller M.C.A., Arbous M.S., Biemond B.J., Blijlevens N.M.A., Kusadasi N., Span L.R.F., Vlaar A.P.J., van Westerloo D.J., Kluin-Nelemans H.C. (2019). Long-Term Outcome of Patients with a Hematologic Malignancy and Multiple Organ Failure Admitted at the Intensive Care. Crit. Care Med..

[36] Vincent F., Soares M., Mokart D., Lemiale V., Bruneel F., Boubaya M., Gonzalez F., Cohen Y., Azoulay E., Darmon M. (2018). In-hospital and day-120 survival of critically ill solid cancer patients after discharge of the intensive care units: Results of a retrospective multicenter study-A Groupe de recherche respiratoire en réanimation en Onco-Hématologie (Grrr-OH) study. Ann. Intensive Care.

[37] Hohenberger P., Buchheidt D. (2005). Surgical interventions in patients with hematologic malignancies. Crit. Rev. Oncol. Hematol..

[38] Mokart D., Darmon M., Resche-Rigon M., Lemiale V., Pène F., Mayaux J., Rabbat A., Kouatchet A., Vincent F., Nyunga M. (2015). Prognosis of neutropenic patients admitted to the intensive care unit. Intensive Care Med..

[39] Theyab A., Algahtani M., Alsharif K.F., Hawsawi Y.M., Alghamdi A., Alghamdi A., Akinwale J. (2021). New insight into the mechanism of granulocyte colony-stimulating factor (G-CSF) that induces the mobilization of neutrophils. Hematology.

[40] Klastersky J., de Naurois J., Rolston K., Rapoport B., Maschmeyer G., Aapro M., Herrstedt J. (2016). Management of febrile neutropaenia: ESMO Clinical Practice Guidelines. Ann. Oncol..

[41] Smith T.J., Bohlke K., Lyman G.H., Carson K.R., Crawford J., Cross S.J., Goldberg J.M., Khatcheressian J.L., Leighl N.B., Perkins C.L. (2015). Recommendations for the Use of WBC Growth Factors: American Society of Clinical Oncology Clinical Practice Guideline Update. J. Clin. Oncol..

[42] National Collaborating Centre for Cancer (2012). National Collaborating Centre for Cancer. National Institute for Health and Care Excellence: Guidelines. Neutropenic Sepsis: Prevention and Management of Neutropenic Sepsis in Cancer Patients.

[43] Cooper A.J., Keller S.P., Chan C., Glotzbecker B.E., Klompas M., Baron R.M., Rhee C. (2020). Improvements in Sepsis-associated Mortality in Hospitalized Patients with Cancer versus Those without Cancer. A 12-Year Analysis Using Clinical Data. Ann. Am. Thorac. Soc..

[44] Kim Y.J., Kang J., Kim M.J., Ryoo S.M., Kang G.H., Shin T.G., Park Y.S., Choi S.H., Kwon W.Y., Chung S.P. (2020). Development and validation of the VitaL CLASS score to predict mortality in stage IV solid cancer patients with septic shock in the emergency department: A multi-center, prospective cohort study. BMC Med..

[45] Azoulay E., Soares M., Darmon M., Benoit D., Pastores S., Afessa B. (2011). Intensive care of the cancer patient: Recent achievements and remaining challenges. Ann. Intensive Care.

