# Supplementary material for: Prognostic Impact of Neutropenia in Cancer Patients with Septic Shock: A 2009–2017 Nationwide Cohort Study
Source: Cancers (Basel). 2022 Jul 24;14(15):3601. doi: 10.3390/cancers14153601 (PMC9332608; doi:10.3390/cancers14153601)
Supplement: Supplementary file 1 [file cancers-14-03601-s001.zip › cancers-1833137-supplementary.pdf]

Supplementary Materials

Prognostic Impact of Neutropenia in Cancer Patients with Septic Shock: A 2009–2017 Nationwide Cohort Study

Sang-Min Kim, Youn-Jung Kim, Ye-Jee Kim and Won-Young Kim

Table S1. An adjusted hazard ratio of neutropenia in 30-day and 1-year mortality in septic shock survivors according to the definition.

Variables	30-Day Mortality			1-Year Mortality		
	**Adjusted HR***	**95% CI**	***p*-Value**	**Adjusted HR***	**95% CI**	***p*-Value**
All malignancy						
Neutropenia code	0.717	0.668–0.770	<0.001	0.773	0.734–0.815	<0.001
Prescription of G-CSF	0.816	0.784–0.850	<0.001	0.864	0.838–0.892	<0.001
Code or prescription	0.811	0.779–0.844	<0.001	0.861	0.836–0.888	<0.001
Only solid cancer						
Neutropenia code	0.695	0.638–0.757	<0.001	0.733	0.688–0.781	<0.001
Prescription of G-CSF	0.849	0.810–0.890	<0.001	0.847	0.817–0.878	<0.001
Code or prescription	0.834	0.796–0.873	<0.001	0.837	0.808–0.867	<0.001

Multivariate analysis included logistic regression analysis and backward elimination. * Adjusted with age (continuous), sex, and charlson comorbidity index. HR, odds ratio; CI, confidential interval; G-CSF, granulocyte-colony stimulating factor.
